# Larvicidal Activity of Essential Oil, Hydrolate, and Aqueous Extract from Leaves of *Myrciaria floribunda* Against *Aedes Aegypti*

**DOI:** 10.3390/molecules30153116

**Published:** 2025-07-25

**Authors:** Eduarda Florencio Santos, Wevertton Marllon Anselmo, Eurico Eduardo Pinto de Lemos, Júlio César Ribeiro de Oliveira Farias de Aguiar, Ana Carla da Silva, Fábio Henrique Galdino dos Santos, Camila Caroline Lopes Arruda, João Vitor Castro Aguiar, José Jorge Almeida de Andrade, Suyana Karolyne Lino da Rocha, Liderlânio de Almeida Araújo, Paulo Gomes Pereira Júnior, Caroline Francisca de Oliveira Albuquerque, Edymilaís da Silva Sousa, Gerlan Lino dos Santos, Tamires Zuleide da Conceição, Leonardo Arcanjo de Andrade, Luiz Alberto Lira Soares, Magda Rhayanny Assunção Ferreira, Daniela Maria do Amaral Ferraz Navarro

**Affiliations:** 1 Department of Chemistry, Center for Exact and Natural Sciences, Federal University of Pernambuco, Recife 50670-901, Brazil; eduarda.florencio@ufpe.br (E.F.S.); wevertton.anselmo@afogados.ifpe.edu.br (W.M.A.); julio.faguiar@ufpe.br (J.C.R.d.O.F.d.A.); carla.silva2@ufpe.br (A.C.d.S.); fabio.henriquegaldino@ufpe.br (F.H.G.d.S.); camila.lopesarruda@ufpe.br (C.C.L.A.); castro.aguiar@ufpe.br (J.V.C.A.); jorge.almeida@ufpe.br (J.J.A.d.A.); suyanarocha@hotmail.com (S.K.L.d.R.); liderlanioalmeida@gmail.com (L.d.A.A.); paulo.gjunior@ufpe.br (P.G.P.J.); oliveira.albuquerque@ufpe.br (C.F.d.O.A.); edymilais@gmail.com (E.d.S.S.); gerlan.lino@ufpe.br (G.L.d.S.); tamires.zuleide@ufpe.br (T.Z.d.C.); leo_arcanjo123@hotmail.com (L.A.d.A.); 2 Engineering and Agricultural Sciences Campus, Federal University of Alagoas, Maceió 57072-900, Brazil; eurico@ceca.ufal.br; 3 Department of Pharmacy, Center of Health Sciences, Federal University of Pernambuco, Recife 50740521, Brazil; luiz.alberto@ufpe.br (L.A.L.S.); magda.raferreira@ufpe.br (M.R.A.F.)

**Keywords:** *Aedes aegypti*, arboviruses, *Myrciaria floribunda*, essential oil, natural products

## Abstract

The mosquito *Aedes aegypti* is the vector responsible for the transmission of important arboviruses such as dengue fever, Chikungunya, Zika virus, and yellow fever. These diseases affect millions of people and exert impacts on healthcare systems throughout the world. Given the increasing resistance to synthetic insecticides, essential oils from plants constitute an ecologically viable alternative for the control of this vector. The aim of the present study was to investigate the larvicidal activity of the essential oil (EO), aqueous extract, rutin, and hydrolate from the leaves of *Myrciaria floribunda* against *Aedes aegypti* larvae in the initial L4 stage. The yield of EO was 0.47%. Thirty-seven chemical constituents were identified and quantified using chromatographic methods. The major constituents were (*E*)-caryophyllene (27.35%), 1,8-cineole (11.25%), β-selinene (4.92%), and α-muurolene (4.92%). In the larvicidal tests, the lethal concentration (LC_50_) was 201.73 ppm for the essential oil, 15.85% for the aqueous extract, and 22.46 ppm for rutin. The hydrolate had no larvicidal activity. The compounds that exhibited larvicidal activity against *Aedes aegypti* constitute a promising option for the development of natural formulations to diminish the propagation of this vector.

## 1. Introduction

Mosquito vectors of arboviruses are strategic targets for disease control actions [1]. *Aedes aegypti* (Linnaeus, 1762) is the main vector responsible for the transmission of arboviruses such as dengue fever, Zika virus, Chikungunya, and yellow fever. This arthropod has spread throughout tropical and subtropical regions and adapts easily to environments that are favorable to its biological cycle, such as places with the availability of clean, still water in artificial containers, as well as adequate food and temperature [2,3].

Dengue fever is one of the fastest-growing infectious diseases globally, reaching 100 to 400 million new infections per year. The disease is currently well established and on the rise in large cities of the tropics [3,4]. Despite investments in large-scale vector control programs in several countries, epidemic outbreaks of arboviruses continue to occur, as the number of mosquitoes resistant to conventional insecticides tends to increase [5,6].

The sustainability of the use of insecticides in vector control programs faces two main obstacles: the emergence of resistance in vectors and the scarcity of new insecticides available on the market [7]. Such limitations related to the use of synthetic insecticides have stimulated interest in conducting research directed at developing safe, effective alternatives, especially natural products from plants [6]. Essential oils and/or plant extracts have demonstrated insecticidal potential in various studies [8,9,10].

The vast diversity of fruit-bearing plants native to Brazil remains underexplored. *Myrciaria floribunda* (H. West ex Willd.) O. Berg is a plant species native to the Atlantic Forest and is also recorded in Central and South America, from southern Mexico to southern Brazil [11,12]. Popularly known as rumberry, *cambuizeiro*, *cambuí*, or guavaberry, this plant produces fruit in a range of colors such as orange, red, and purple [13]. The essential oil extracted from the leaves of *M. floribunda* has various terpene compounds, conferring considerable bioactive potential to the plant [14,15].

The literature reports the antioxidant potential of the essential oil from *M. floribunda* (Myrtaceae) [15] and that the phenolic compounds, flavonoids, and tannins in the leaf extract of this plant have antinociceptive and anti-inflammatory potential [16], as well as antiproliferative activity in tumor cells [17]. Other species of the family Myrtaceae are widely known due to their biological activities against *Ae. aegypti*. For instance, *Eugenia uniflora* [18], *Eugenia gracillima* [19], and *Eugenia estipitata* [20] have demonstrated significant larvicidal activity against this vector.

According to Simas and collaborators [21], sesquiterpenes exhibit greater larvicidal activity compared to monoterpenes against *Ae. aegypti* larvae due to the lipophilic properties of terpenes, which enhance transmembrane absorption by the organism and may cause toxic effects; sesquiterpenes are the most active in this regard.

Previous studies, such as that conducted by Tietbohl and collaborators [10], indicated that the essential oil extracted from *M. floribunda* is composed predominantly of sesquiterpenes, followed by monoterpenes, and exhibits insecticidal activity. Significant activity was found against *Rhodnius prolixus*, which is the vector of Chagas disease, with increased mortality and interference with the metamorphosis process of the insect.

The selection of *M. floribunda* for the present study is therefore justified both by the insecticidal potential of the essential oil previously demonstrated in the literature and the lack of studies on the larvicidal activity of the essential oil, aqueous extract, and hydrolate from the leaves of this species against *Ae. aegypti* larvae. Investigating this gap contributes to the search for novel vector control options, especially based on native Brazilian plant species with bioactive potential, which is not yet fully understood.

Therefore, the aim of the present study was to investigate the larvicidal activity of the essential oil (EO), aqueous extract, and hydrolate from the leaves of *Myrciaria floribunda* against *Ae. aegypti* larvae in the initial L4 stage.

## 2. Results

### 2.1. Yield and Chemical Identification of Essential Oil from Leaves of M. floribunda

In the experimental tests, the yield of the essential oil from leaves of *M. floribunda* was 0.47%. The chromatographic analysis revealed 61 peaks. Thirty-seven compounds were identified (Table 1). The chromatogram is available in the supplementary material (Figure S1).

The major compounds were (E)-caryophyllene (27.35%), 1,8-cineole (11.25%), β-selinene (4.92%), and α-muurolene (4.92%). The essential oil is composed of the terpene class, with a predominance of sesquiterpenes (69.61%), followed by monoterpenes (17.59%), as shown in Table 1. Terpenes constitute one of the largest groups of natural bioactive compounds, encompassing monoterpenes, sesquiterpenes, diterpenes, hemiterpenes, and triterpenes [16].

### 2.2. Chemical Characterization of Aqueous Extract

**Figure 1:**
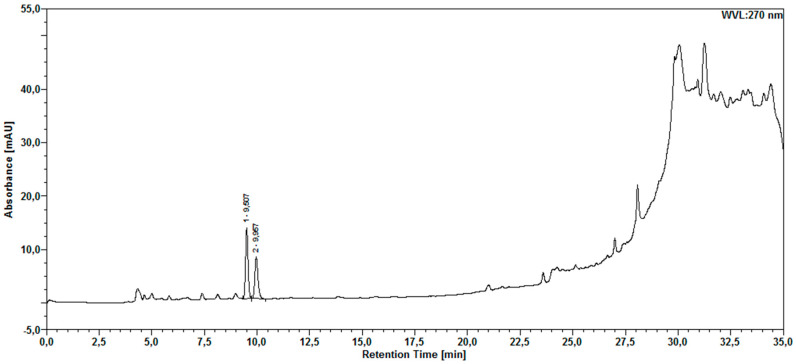
Chromatogram of aqueous extract sample with detection at 270 nm.

**Figure 2:**
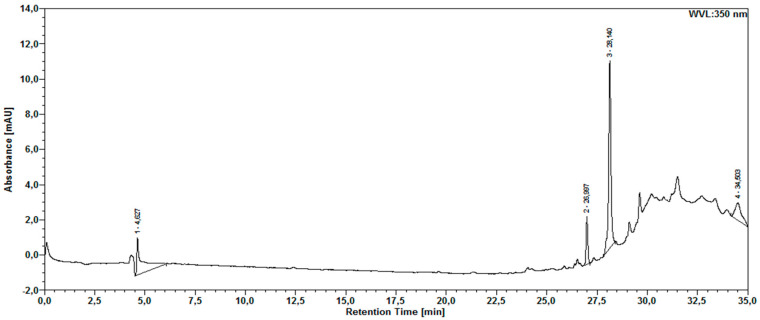
Chromatogram of aqueous extract sample with detection at 350 nm.

**Figure 3:**
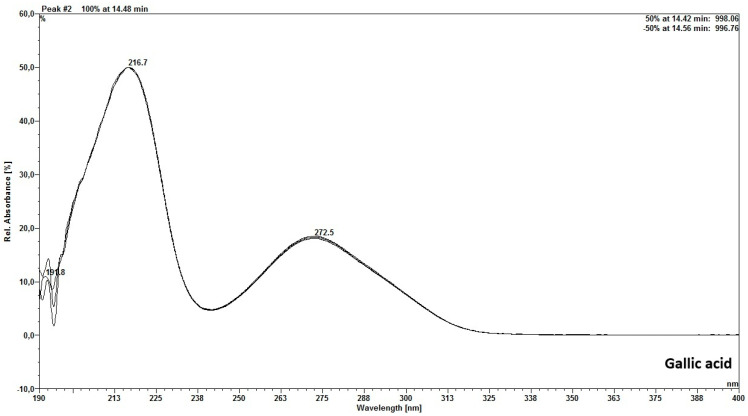
Scanning spectrum of peak similar to gallic acid standard.

**Figure 4:**
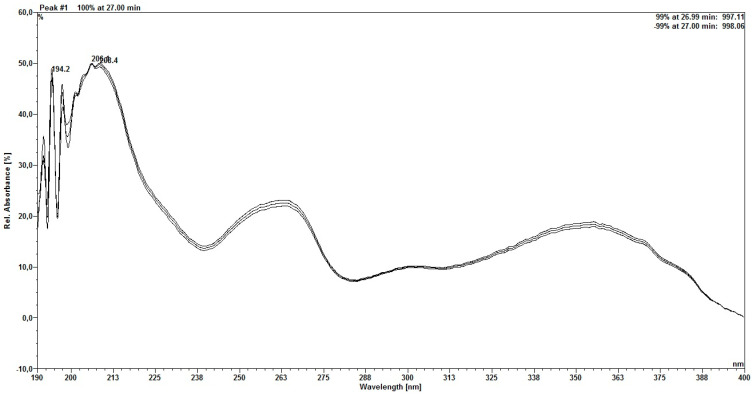
Scanning spectrum of first peak found for rutin.

The chromatographic analysis of the aqueous extract by HPLC-DAD revealed compounds detected at 270 nm (Figure 1) and 350 nm (Figure 2). Two secondary metabolites were also found in the *M. floribunda* extract, which have a scanning spectrum similar to that of the gallic acid and rutin standards (Figure 3 and Figure 4).

It was not possible to identify all substances in the aqueous extract. Using the DAD detector, however, the analysis of the UV scanning spectra indicated the presence of two flavonoids at 350 nm of the chromatogram.

The DAD detector revealed peaks with UV scanning spectra and retention times similar to the gallic acid standard (Figure 3), corresponding to the presence of this compound at Rt = 9.96 min, with maxima at 216.4/270 nm.

**Figure 5:**
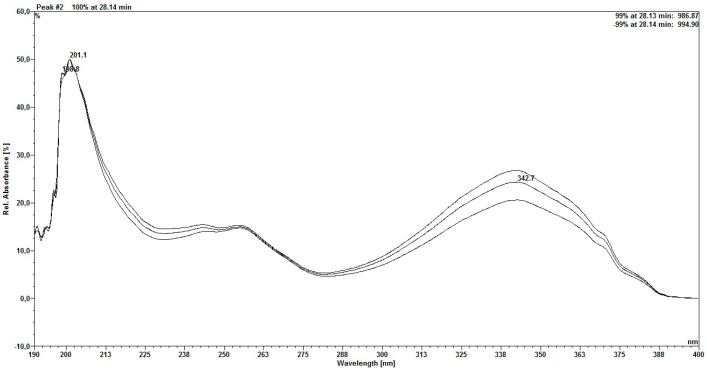
Scanning spectrum of the second peak found for rutin.

The first peak has a scanning spectrum similar to that of the rutin standard (Figure 4), which is a glycosylated flavonol, whose absorption maxima occur at 256.3/356.8 nm. The second peak (Figure 5) has absorption maxima at 257/342.7 nm, which is considered characteristic of a flavonoid [22].

### 2.3. Larvicidal Activity

The results of the larvicidal assay are displayed in Table 2 and Tables S1–S3 of the Supplementary Materials. The tables in the Supplementary Materials describe the concentrations tested, the total number of larvae exposed in each bioassay, the total number of larvae killed per concentration, and the respective mortality rates after 48 h of exposure [18]. Table 2 displays the LC_50_ values for the essential oil from the leaves of *M. floribunda* (LC_50_ = 201.73 ppm), aqueous extract (LC_50_ = 15.85% (*v*/*v*)), and rutin (LC_50_ = 22.09 ppm). The hydrolate did not cause larval mortality and was therefore not included in the results table. No larval mortality was recorded in the negative control test with Tween 80, whereas 100% mortality was found using Temephos at 1 ppm as the positive control.

## 3. Discussion

### 3.1. Yield and Chemical Characterization of Essential Oil from M. floribunda

The yield of the essential oil (0.47%) was higher than that reported by Tietbohl and collaborators [10], who found a yield of 0.37% after four hours of extraction. In contrast, Barbosa and collaborators [23] reported a yield of 0.60% using the fruit peels of *M. floribunda*. In another study, a yield of 1.02% was reported for the EO extracted from dried leaves. This divergence suggests that the oil content may be influenced by the type and state of the plant matrix [15]. Tietbohl and collaborators [14] found that the yield varied according to the part of the plant analyzed (0.37% for leaves, 0.02% for stems, and 0.64% for flowers). Thus, the yield obtained in the present study is within the range reported in the literature.

The chemical composition of the essential oil from *M. floribunda* in this study is in agreement with data described in the literature. For instance, Moraes and collaborators [15] collected the species from the city of Bujaru (state of Pará, Brazil) and found the major constituents to be 1,8-cineole (23.30%), terpinolene (22.23%), and α-phellandrene (22.19%). Tietbohl and collaborators [14] analyzed the essential oil extracted from the leaves of *M. floribunda* collected from Jurubatiba Sandbank National Park (state of Rio de Janeiro, Brazil) and identified a chemical profile characterized mainly by monoterpenes (53.9%), especially 1,8-cineole, which was the major compound (38.4%). Although 1,8-cineole was found in greater quantities in these previous studies, its occurrence as one of the major compounds in the present investigation confirms its representativeness among the main chemotypes of the species.

Tietbohl and collaborators [10] identified 1,8-cineole (10.4%), β-selinene (8.4%), α-selinene (7.4%), selin-11-em-4-α-ol (4.3%), and α-trans-bergamotene (3.9%) as the main chemical constituents in the essential oil. In more recent studies, the authors characterized the essential oil from the leaves of *M. floribunda*, reporting the following major constituents: nerolidol (15.4%), β-selinene (13.9%), 1,8-cineole (10.7%), and zonarene (7.67%) [24]. These data demonstrate the occurrence of different chemotypes with a predominance of the monoterpene 1,8-cineole, which was also one of the main compounds identified in the present study.

Studies have investigated the chemical composition of the fruit peels of *M. floribunda* and identified the presence of volatile organic compounds, with γ-selinene (58.18%), caryophyllene (48.51%), patchoulene (32.56%), α-longipinene (24.21%), α-muurolene (21.04%), and 1,8-cineole (10.6%) as the major compounds [13]. The following compounds were identified in the EO extracted from the fruit peel: δ-cadinene (26.8%), γ-cadinene (15.69%), γ-muurolene (6.21%), α-selinene (6.11%), α-muurolene (6.11%), and (E)-caryophyllene (5.54%) [23]. These findings are in agreement with the chemical constituents identified in the present investigation. The chemical composition of the EO from *M. floribunda* leaves may vary depending on the plant collection site and the extraction method employed, resulting in different chemotypes. This qualitative and quantitative variability may be attributed to intrinsic factors of the plant, such as soil type, climate, and degree of maturity, as well as extrinsic factors, such as the extraction method, environmental conditions, and even the time of day when the plant was collected [25].

### 3.2. Chemical Characterization of Aqueous Extract

The chemical characterization of the extract revealed the presence of two secondary metabolites: gallic acid and rutin. In the study conducted by Tietbohl and collaborators [11], these same metabolites were quantified in the ethyl acetate extract of *M. floribunda* leaves. The wide applicability of species of the family Myrtaceae can be attributed to phytochemical constituents, specifically flavonoids [26]. Flavonoids originate from the secondary metabolism of plants and have vast biological activity, with antioxidant, anti-inflammatory, antibacterial, antiallergic, and vasodilatory properties [16].

Gallic acid is found in most plants and is a benzoic acid of considerable relevance in the formation of the group of hydrolyzable tannins known as gallotannins [27]. Studies involving the extracts of other species belonging to the family Myrtaceae, such as *Eugenia involucrata* DC, identified the presence of gallic acid in the seeds of the fruits [28] and rutin in the leaves of the species [29]. Gallic acid was also identified in the aqueous extract from *E. uniflora* (Myrtaceae) [18].

### 3.3. Larvicidal Activity

The World Health Organization does not establish specific values to determine the efficacy of a compound or extract in combating insects [30]. The literature suggests parameters for assessing the larvicidal potential of essential oils against *Aedes aegypti* larvae at the L4 stage. Classifications are established based on the median lethal concentration (LC_50_) of the EO: LC_50_ > 100 ppm is considered low or inactive; LC_50_ < 100 ppm is classified as active; and LC_50_ < 50 ppm indicates high larvicidal activity [31]. The present results reveal greater effective larvicidal activity against *Ae. aegypti* compared to essential oils from plant species endemic to the Amazon belonging to the family Myrtaceae, such as *Eugenia piauhiensis* Vellaff. (LC_50_ = 230 ppm) and *Myrcia erythroxylon* O. Berg (LC_50_ > 1000 ppm) [32]. Studies conducted with the EO from the leaves of *M. floribunda* whose major constituent is 1,8-cineole demonstrated bioinsecticidal activity against the pests *Oncopeltus fasciatus* (LD_50_ = 112.44 µg/insect) and *Dysdercus peruvianus* (LD_50_ = 309.64 µg/insect) after 24 h of exposure [14]. Three EOs from species of eucalyptus (Myrtaceae) whose major constituent is 1,8-cineole demonstrated insecticidal activity against larvae and engorged females of *B. microplus* [33].

As the evaluated essential oil showed an LC_50_ value of 201.73 ppm, it was decided not to proceed with testing its major constituents, since the EO demonstrated low biological efficacy according to the parameters established by Cheng and collaborators [31]. However, the major constituent identified in this study, (*E*)-caryophyllene (27.35%), which is found in various essential oils—such as the essential oil of *Commiphora leptophloeos*, which contains 18% (*E*)-caryophyllene—is reported to exhibit larvicidal and oviposition-deterrent effects against *Ae. aegypti* [34]. Moreover, essential oils from Croton linearis (1.28% (*E*)-caryophyllene), *Lantana involucrata* (13.04% (E)-caryophyllene), *Ocimum sanctum var. cubensis* (17.85% (E)-caryophyllene), and *Zanthoxylum pistaciifolium* Griseb. (3.06% (*E*)-caryophyllene) have also been reported to exhibit larvicidal and adulticidal activity against *Ae. aegypti*, *Anopheles albitarsis*, and *Culex quinquefasciatus* [35].

Different species of the family Myrtaceae have significant larvicidal activity. However, few studies have investigated this potential in the byproducts of hydrodistillation, such as the aqueous extract and hydrolate. Silva and collaborators [18] reported larvicidal activity against *Ae. aegypti* using the aqueous extract (LC_50_ = 12.205 ± 1.04 mg/L) and hydrolate (LC_50_ = 42.4 ± 1.02 mg/L) of the species *Eugenia uniflora* (Myrtaceae).

In the present study, the aqueous extract exhibited larvicidal activity (LC_50_ = 15.85% (*v*/*v*)). The potential for larvicidal activity may vary considerably among species, even within the same botanical family, due to distinct phytochemical profiles. There are no records of previous studies on the aqueous extract from *M. floribunda* leaves for larvicidal activity against *Ae. aegypti*. Therefore, the present investigation is innovative, demonstrating that this species is a promising source for the formulation of novel natural insecticides and contributing to the expansion of knowledge on its bioactive potential in the control of *Ae. aegypti*.

As shown in Table 2, rutin exhibited larvicidal activity, with LC_50_ = 22.46 ppm, demonstrating greater efficiency in comparison to results of the study conducted by Guarda and collaborators [36], in which the mortality of *Ae. aegypti* larvae in the L2 and L3 stages required concentrations of 500, 750, and 1000 ppm in 48 h. In another study, this flavonoid had negative effects on the larval development of the species *Spodoptera frugiperda*, prolonging larval development time, reducing the weight of larvae and pupae, and diminishing pupal viability [37]. Recent experiments indicated the significant inhibitory effect of rutin on the growth and development of *Bacillus thuringiensis* (Bt)-susceptible and Bt-resistant strains of the pink bollworm (*Pectinophora gossypiella*) [38].

Flavonoids are polyphenolic compounds belonging to the group of secondary metabolites produced by plants [39]. The present results underscore the importance of studies on products extracted from plant matrices, highlighting their potential as promising options for the production of natural insecticides for the control *Ae. aegypti*, especially in the larval stage.

Lima and collaborators [40] investigated the effects of the EO from *Piper tuberculatum* and its major compound (β-caryophyllene; 54.8% of the oil) on *Ae. aegypti* larvae, as well as toxicity to non-target organisms. Both treatments exhibited significant larvicidal activity (LC_50_ of 48.61 and 57.20 ppm, respectively; *p* < 0.05), the inhibition of the enzyme acetylcholinesterase (IC_50_ of 57.78 and 71.97 ppm), and increased production of reactive oxygen and nitrogen species in the larvae after exposure to the EO and β-caryophyllene. Therefore, acetylcholinesterase inhibition is a possible mechanism of action that may explain the larval mortality observed for the essential oil in the present study. The mode of action of the compound rutin on *Ae. aegypti* larvae has not been fully clarified in the literature, underscoring the need for further studies to broaden this understanding.

Based on the study by Silva and collaborators [18], the contribution of gallic acid to the biological activity against *Ae. aegypti*, as observed in the aqueous plant extract, is not significant. The authors suggested that the pronounced biological activity found against the mosquito was related to the major volatile compounds present in the plant. In a preliminary study conducted by our research group, we also found that gallic acid does not exhibit larvicidal activity against *Ae. aegypti* larvae. Therefore, its effect may be limited or dependent on synergistic interactions with other bioactive compounds in the aqueous extract.

## 4. Materials and Methods

### 4.1. Plant Material and Extraction of Essential Oil

Fresh leaves of the plant *M. floribunda* (West ex Willd.) O. Berg were obtained from the Cambuizeiro Active Germplasm Bank at the Center of Agricultural Sciences of the Federal University of Alagoas in the municipality of Rio Largo, state of Alagoas, Brazil (latitude 9°29′45″ S, longitude 35°49′54″ W; Sisgen number: A668DC0). The plant material was taken to the Chemical Ecology Laboratory of the Department of Fundamental Chemistry of the Federal University of Pernambuco in the city of Recife, state of Pernambuco, Brazil, where it was submitted to hydrodistillation to obtain the essential oil and aqueous extract.

Fresh leaves (800 g) of *M. floribunda* were ground in a blender, transferred to a 5 L round-bottom flask, and submitted to hydrodistillation with three liters of distilled water for three hours using a Clevenger apparatus. The essential oil was dried using anhydrous sodium sulfate (Na_2_SO_4_), subsequently transferred to appropriate containers (vials), and kept refrigerated at −4 °C. The by-products resulting from the hydrodistillation process (aqueous extract and hydrolate) were also properly stored and kept refrigerated until the biological tests. The yield of the oil was calculated by dividing the mass of the essential oil by the mass of the fresh leaves (*w*/*w*). Rutin was purchased commercially.

### 4.2. Identification of Essential Oil

The composition of the essential oil was identified by gas chromatography–mass spectrometry (GCMS) using an Agilent 5975C series GC/MSD system (Agilent Technologies, Palo Alto, CA, USA) equipped with a quadrupole and an unpolished fused silica DB-5 capillary column (30 m × 0.25 mm i.d. and film thickness of 0.25 μm) (Agilent Technologies, Palo Alto, CA, USA). A 1 μL sample of the hexane solution of the oil (100 ppm) was injected into the split-mode injector (50:1), with the temperature maintained at 250 °C. The oven temperature started at 40 °C for 2 min, followed by an increase of 4 °C/min until reaching 230 °C, where it remained for 5 min. The flow rate of helium gas used as the mobile phase was kept constant at 1 mL/min, with a pressure of 7.0 psi. The MS source and quadrupole temperatures were set at 230 °C and 150 °C, respectively [41]. Mass spectra were obtained at 70 eV (electron impact ionization mode), with a scanning rate of 1.0 s, encompassing the *m*/*z* 35–350 range [42].

Chemical constituents were identified by comparing mass spectra with retention indices obtained by co-injecting essential oil samples with a homologous series of hydrocarbon standards (C9–C30, Sigma-Aldrich) calculated according to the Van den Dool and Kratz equation [43]. The mass spectra of each chemical constituent of the essential oil were compared by determining similarities with spectra available in GC-MS libraries (NIST08; WILEY7N; ESSENTIALOILS-23P) and validated with mass spectral data available in the literature [44].

Quantification of the constituents of the essential oil was performed using a gas chromatograph equipped with a flame ionization detector (GC-FID) (Thermo Trace GC Ultra, Milan, Italy), with the detector temperature set at 250 °C and a nonpolar VB-5 column (Thermo Trace GC Ultra, 60 m × 0.25 mm i.d.; film thickness of 0.25 μm). A 1.0 μL aliquot of the hexane solution (100 ppm) was injected in triplicate in splitless mode under the same conditions as described for GCMS.

### 4.3. Characterization of Non-Volatile Secondary Metabolites of Aqueous Extract

The chemical characterization of the aqueous extract from *M. floribunda* leaves was performed in partnership with the Pharmacognosy Laboratory of the Department of Pharmaceutical Sciences of the Federal University of Pernambuco in the city of Recife, state of Pernambuco, Brazil. The aqueous extract was analyzed for the presence of non-volatile secondary metabolites. The sample was prepared at 1 mg/mL in a 25 mL flask, completing the volume with ultrapure water (Purelab Classic UV, Elga^®^, High Wycombe, UK). The sample was then filtered into vials with the aid of a PVDF filter (0.45 µm; Chromafil^®^, Higashiosaka City, Japan). The gallic acid standard (≥94%, Sigma-Aldrich^®^, Burlington, MA, USA) was prepared in ultrapure water (Purelab Classic UV, Elga^®^), and the resulting solution was filtered into vials with the aid of a PVDF filter (0.45 µm; Chromafil^®^).

High-performance liquid chromatography was performed using an Ultimate 3000 HPLC system (Thermo Fisher Scientific, Waltham, MA, USA) coupled to a photodiode array detector (DAD; Thermo Fisher Scientific) and equipped with a binary pump (HPG-3x00RS, Thermo Fisher Scientific), degasser, and autosampler equipped with a 20 µL loop (ACC-3000, Thermo Fisher Scientific). The wavelengths were 270 and 350 nm. Chromatographic separations were obtained with a C18 column (250 mm × 4.6 mm i.d., 5 µm; Supelco^®^, Burlington, MA, USA) equipped with a pre-column (C18 4 mm × 3.9 µm; Phenomenex^®^, Lane Cove, NSW, Australia). Separations were performed at 24 ± 1 °C. The mobile phase consisted of ultrapure water (A) and methanol (B), both acidified with 0.05% trifluoroacetic acid, with the flow adjusted to 0.9 mL/min. A gradient program was applied: 0–10 min, 10–2% B; 10–13.5 min, 20–25% B; 13.5–18 min, 25–40% B; 18–25 min, 40–80% B; 25–30 min, 80% B; 30–35 min, 80–10% B. The data were analyzed after injection in triplicate and processed using the Chromeleon 6.8 software program (Dionex/Thermo Fisher Scientific, USA).

### 4.4. Aedes Aegypti Colony

To perform the bioassays, larvae were used from the *Ae. aegypti* Linneaus (Rockefeller strain) colony maintained at the Bioassay Laboratory of the Department of Fundamental Chemistry of the Federal University of Pernambuco at 27 ± 1 °C and relative humidity of 75 ± 1%. The rearing of the colony involved the initial stage of hatching of the *Ae. aegypti* eggs, on which cards were placed in rectangular plastic basins with distilled water to cover the eggs sufficiently, and small amounts of ground Whiskas^®^ cat food were added. During the larval development stage, larvae were distributed in other plastic basins with distilled water and food. Development and maintenance of the larvae were monitored, with the changing of the distilled water and addition of food every two days, until the larvae reached the initial L4 stage, which was a necessary step for the larvicidal bioassays.

### 4.5. Larvicidal Bioassays

Preliminary tests were performed prior to the larvicidal test with the *M. floribunda* essential oil. A stock solution was prepared at a concentration of 100 ppm, weighing 5 mg of the essential oil and adding 3 drops of Tween 80 (co-solvent). After homogenization, the material was transferred to a 50 mL volumetric flask and completed with distilled water. Preliminary tests were performed at concentrations of 10 ppm, 50 ppm, and 100 ppm, with the aim of determining the concentration range that encompassed the best larvicidal activity of the essential oil. For each larvicidal bioassay, 20 L4 larvae were distributed among 40 mL beakers containing a final volume of 20 mL for each test concentration.

The negative control was performed in triplicate using distilled water and Tween 80^®^, and no larval mortality was observed. The approximate mass of 3 drops of Tween 80 (density of 1.06 g/mL) was 2.6 mg for a 50 mL volumetric flask, corresponding to a concentration of 52 ppm or 0.24% (*v*/*v*), which is too low to cause mortality, as experimentally observed in the negative control and reported in the literature by Kramer and collaborators [45], who found Tween 80 toxicity to be LC_50_ = 8%, *v*/*v*. A Temephos solution at 1 ppm was used as the positive control. Larval mortality was determined after 24 and 48 h of immersion in the concentrations tested. Larvae that did not respond to stimuli or did not rise to the surface of the solution were considered dead [46].

Concentrations that resulted in mortality between 20% and 80% in the preliminary tests were selected. The bioassay for the *M. floribunda* essential oil was performed in triplicate to determine the LC_50_ values using concentrations of 80, 100, 150, 180, 200, and 250 ppm.

For the larvicidal tests with the byproducts of hydrodistillation, the aqueous extract and hydrolate were diluted in distilled water alone, resulting in concentrations (*v*/*v*) of 5%, 7.5%, 10, 15, 20, 25, 30, and 35% (*v*/*v*) and 100, 150, and 200% (*v*/*v*), respectively, in a final volume of 20 mL. Rutin (compound characterized in the aqueous extract) was dissolved in three drops of Tween 80 (co-solvent), and distilled water was added until completing the 250 mL flask. The following concentrations were tested: 20, 25, 30, 35, and 40 ppm. The LC_50_ (lethal concentration that kills 50% of the larvae) was calculated using the Pro 6.2.5.0 statistical survival analysis software with a 95% confidence level [18].

## 5. Conclusions

This study demonstrated the larvicidal potential of the essential oil from the leaves of *Myrciaria floribunda* against *Ae. aegypti* larvae. The yield was 0.47%, and the essential oil exhibited moderate larvicidal activity (LC_50_ = 201.73 ppm). The oil was characterized by a predominance of sesquiterpenes, with (*E*)-caryophyllene as the major component. The aqueous extract proved to be the most effective (LC_50_ = 15.85% *v*/*v*), followed by isolated rutin (LC_50_ = 22.46 ppm), which is a flavonoid identified in the extract along with gallic acid. The hydrolate exhibited no larvicidal activity. The results demonstrate the promising biological activity of the aqueous extract and rutin, highlighting their potential as natural alternatives for controlling the *Ae. aegypti* vector. This study also stands out by valuing the by-products of hydrodistillation, thus contributing to the sustainable use of plant-derived bioactive compounds. The present findings confirm the importance of investigating natural products as viable strategies to be integrated into vector management programs.

## 6. Patents

This article has intellectual property rights, with a national patent application for invention, utility model, certificate of addition of invention, and entry into the national phase of the Patent Material Cooperation Agreement with the National Institute of Industrial Property (INPI) under process number BR 10 2024 014794 4.

## Figures and Tables

**Table 1:** Chemical composition of essential oil from leaves of *M. floribunda*.

Chemical Constituent ^a^	RI-Calculated ^b^	RI-Literature ^c^	% ^e^	SD ^d^
α-Thujene	923	924	0.17	0.01
α-Pinene	929	932	2.30	0.17
Camphene	943	946	0.37	0.02
β-Pinene	972	974	0.14	0.01
Myrcene	990	988	0.51	0.03
α-Phellandrene	1001	1002	1.60	0.11
α-Terpinene	1014	1014	0.19	0.02
ρ-Cymene	1022	1020	0.17	0.02
1.8-Cineole	1029	1026	11.25	0.89
(E)-β-Ocimene	1048	1044	0.18	0.02
γ-Terpinene	1057	1054	0.48	0.04
Terpinolene	1086	1086	0.23	0.01
α-Cubebene	1350	1348	0.78	0.04
α-Ylangene	1372	1373	0.25	0.01
α-Copaene	1376	1374	2.30	0.16
Sativene	1390	1390	0.17	0.01
α-Gurjunene	1410	1409	0.84	0.03
E-Caryophyllene	1422	1417	27.35	0.56
β-Copaene	1430	1430	1.77	0.13
Aromadendrene	1440	1439	1.37	0.18
α-Humulene	1455	1452	3.26	0.05
Allo-aromadendrene	1462	1458	1.62	0.04
Trans -1(6).4-diene-cadina	1475	1475	2.53	0.13
γ-Muurolene	1478	1478	0.61	0.13
α-Amorphene	1481	1483	2.34	0.04
β-Selinene	1487	1489	4.92	0.17
δ-Selinene	1492	1492	061	0.13
Viridiflorene	1496	1496	2.34	0.04
α-Muurolene	1501	1500	4.92	0.17
δ-Amorphene	1509	1511	2.73	0.06
γ-Cadinene	1512	1513	1.56	0.04
δ-Cadinene	1526	1522	3.41	0.08
Zonarene	1528	1528	1.34	0.08
Trans-cadina-1.4-diene	1534	1533	0.48	0.00
α-Cadinene	1539	1537	0.44	0.05
α-Calacorene	1544	1544	1.23	0.08
Caryolan-8-ol	1571	1571	0.43	0.07
Total identified			87.20	
Not identified			12.8	
Total monoterpenes			17.59	
Total sesquiterpenes			69.61	

^a^ Constituents listed in order of elution in nonpolar DB-5 column; ^b^ retention index calculated based on retention times of n-alkane series (C_9_—C_30_); ^c^ retention index from the literature; ^d^ SD: standard deviation; ^e^ %: area of constituent relative to essential oil.

**Table 2:** Larvicidal activity of essential oil and aqueous extract from leaves of *M. floribunda* and rutin against *Ae. Aegypti* larvae after 48 h of exposure.

Components Tested	N ^a^	DF ^b^	LC_50_ (95% CI) ^c,d^ (LLC-ULC) ^e^	LC_90_ (95% CI) ^c,d^ (LLC-ULC) ^e^	X^2 e^	Slope (SE)
Essential oil	660	3	201.73 ± 8.73 ppm (172.84–230.62)	604.78 ± 1.05 ppm (456.72–957.42)	0.30	0.33
Aqueous extract	400	3	15.85 ± 0.2 % (15.02–16.69)	19.92 ± 1.05% (20.06–34.21)	0.004	0.79
Rutin	420	3	22.46 ± 0.99 ppm (19.17–25.75)	43.55 ± 1.08 (38.52–53.60)	0.44	0.63

^a^ Number of larvae used in test; ^b^ degrees of freedom; ^c^ lethal concentration and confidence interval; ^d^ calculations using StatPlus Pro 6.2.5.0 statistical software; ^e^ minimum and maximum lethal concentrations estimated by statistician.

## Data Availability

All data are found in the article itself.

## Supplementary Materials

The following supporting information can be downloaded at: https://www.mdpi.com/article/10.3390/molecules30153116/s1, Figure S1: Chromatogram of *Myrciaria floribunda* essential oil; Table S1: Results of larvicidal assay with essential oil from leaves of *Myrciaria floribunda* oil; Table S2: Results of larvicidal assay with aqueous extract *Myrciaria floribunda*; Table S3: Results of larvicidal assay with the compound rutin.
