# Supplementary material for: Larvicidal Activity of Essential Oil, Hydrolate, and Aqueous Extract from Leaves of Myrciaria floribunda Against Aedes Aegypti
Source: Molecules. 2025 Jul 25;30(15):3116. doi: 10.3390/molecules30153116 (PMC12348816; doi:10.3390/molecules30153116)
Supplement: Supplementary file 1 [file molecules-30-03116-s001.zip › molecules-3733431-supplementary.pdf]

Supplementary Material:

Characterization of the essential oils of *Myrciaria floribunda*

Figure S1: Chromatogram of the oil essential of Myrciaria floribunda

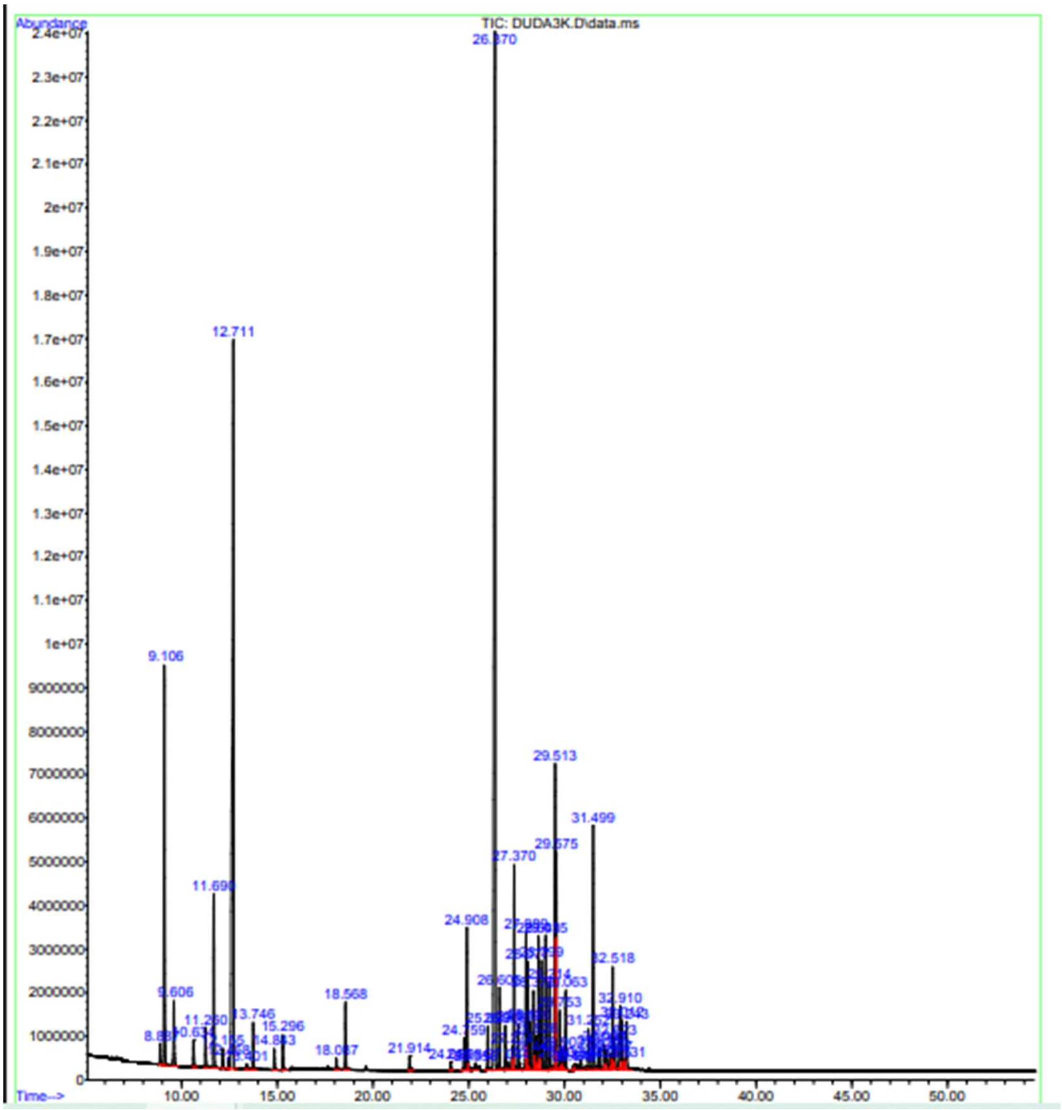

Larvicidal activity: Tables S1 to S3 refer to the experimental results of the larvicide bioassays, the total number of dead larvae and the total number of larvae used at each concentration and the mortality percentage for each concentration range used in determining the LC50.

Table S1: Results of larvicidal assay with essential oil from leaves of *Myrciaria floribunda* essential oil;

Concentration	Total no. of dead	Total no. of larvae	% mortality
80	7	60	11.67
100	39	160	24.38
150	43	120	35.83
180	46	100	46
200	61	120	50.83
250	61	100	61

Table S2: Results of larvicidal assay with aqueous extract *Myrciaria floribunda*

Concentration	Total no. of dead	Total no. of larvae	% mortality
10	13	80	16
15	23	60	38
16	42	80	53
17	39	60	65
18	45	60	75
19	50	60	83

Table S3: Results of larvicidal assay with the compound rutin

**Concentration**	**Total no. of dead**	**Total no. of larvae**	**% mortality**
20	53	120	44.17
25	34	60	56.67
30	85	120	70.83
35	46	60	76.67
40	55	60	91.67
